# Total ankle replacement through a lateral approach: surgical tips

**DOI:** 10.1051/sicotj/2016029

**Published:** 2016-11-18

**Authors:** Federico Giuseppe Usuelli, Cristian Indino, Camilla Maccario, Luigi Manzi, Vincenzo Salini

**Affiliations:** 1 CASCO, IRCCS Istituto Ortopedico Galeazzi via Riccardo Galeazzi 4 20161 Milan Italy; 2 Scuola di Specializzazione in Ortopedia e Traumatologia, Universita’ degli Studi di Milano Via della Commenda 10 20122 Milan Italy; 3 Orthopaedics and Traumatology, University G. dAnnunzio, Chieti Pescara Via dei Vestini 66100 Chieti Italy

**Keywords:** Total ankle replacement, Ankle osteoarthritis, Lateral approach

## Abstract

*Purpose*: Recently, the Zimmer Trabecular Metal Total Ankle Replacement (Zimmer TM TAR) was developed to be used through a lateral transfibular approach. The purpose of this paper is to describe the surgical technique and early outcomes of the TAR via the lateral approach using the Zimmer TM TARs.

*Methods*: Sixty-seven patients underwent primary TAR using the Zimmer TM TAR between May 2013 and May 2015. Patients were clinically evaluated preoperatively and postoperatively at six and twelve months and annually using the American Orthopaedic Foot & Ankle Society (AOFAS) ankle and hindfoot scores, visual analogue scale (VAS) pain score, and the Short Form Health Survey (SF-12) questionnaire. The minimum follow-up was 12 months.

*Results*: The mean AOFAS hindfoot score increased from 32.8 preoperatively to 85.0 at the latest follow-up (*p*-value < 0.001). The mean VAS pain score decreased from 8.0 to 2.0 at the latest follow-up (*p*-value < 0.001). The Physical and Mental Health Composite Scale scores (PCS and MCS) of the SF-12 passed from a mean value of 30.2 preoperatively to 43.1 (*p*-value < 0.001) and from a mean value of 44.6 to 53.5 at the latest follow-up (*p*-value < 0.001), respectively.

*Conclusions*: We present our surgical tips and the early results of this prosthetic design which are encouraging. They could be useful as an adjunct to the manufacturer’s surgical technique guidance for surgeons who utilize these implants.

## Introduction

Ankle arthrodesis and total ankle replacement (TAR) are standard procedures for ankle osteoarthritis (AO) when conservative treatment has failed [1]. Long-term data concerning ankle arthrodesis showed important disadvantages: compensatory overload, gait change, high rates of non-union, long rehabilitation period, and development of adjacent joint arthritis [1, 2]. The number of TARs being performed is increasing because of the availability of new implant design with the possibility of saving tibio-talar range of motion (ROM) and preventing adjacent joints’ degeneration [3]. Encouraging reports of mid- to long-term success of last-generation TAR continue to emerge, as techniques for the use of these devices become better defined [4].

Traditionally, TARs have been performed through an anterior approach that allows for an optimal visualization of the joint in the coronal plane, but it is subject to soft-tissue complications [5, 6]. Recently, a new TAR design (Zimmer Trabecular Metal Total Ankle, Zimmer, Warsaw, IN) was developed to be used through a lateral transfibular approach. This approach provides direct visualization of the center of rotation of the ankle, allowing for more accurate reconstruction of the joint alignment as well as less bone resection with anatomically curved resections of the talus and plafond. Additionally, it allows the implants to be placed perpendicular to the trabeculae of the tibia and talus, decreasing the shear forces at the bone-implant interface [7]. Zimmer Trabecular Metal (TM) TAR is a two-component, fixed-bearing device with a highly cross-linked polyethylene on metal-bearing surface. It requires an alignment external frame and milling device for accurate insertion. The alignment stand is a rigid coordinate system to base the bony resections and it enables correction of sagittal and coronal deformities, theoretically improving the reliability of the positioning of the implants.

The purpose of this paper is to describe the surgical technique and early outcomes of TAR via lateral approach using Zimmer TM TARs.

## Material and methods

### Study design

The study was approved by our Institutional Review Board and it has been performed in accordance with the ethical standards laid down in the 1964 Declaration of Helsinki and its later amendments. Between May 2013 and May 2015, 67 patients underwent primary TAR using the Zimmer TM TAR through a lateral transfibular approach in our institution. All the procedures were performed by the senior author. Thirty-seven patients (55.2%) were men and 30 (44.8%) were women. The mean age at the time of surgery was 50.5 years (standard deviations *SD* 13.1, range 21–78). The mean follow-up was 15 months (*SD* 6.0, range 12–36). The indications for TAR included primary degenerative osteoarthritis, systemic (rheumatoid) arthritis, and secondary osteoarthritis (e.g., posttraumatic arthritis, hemophilia, hereditary hemochromatosis, gout, and postinfectious arthritis). Neuropathic arthropathy, neuromuscular disorders, pathologic joint laxity, acute infectious arthritis, and avascular necrosis of the talus involving greater than 50% of the bone were contraindications for TAR [8]. The clinical data were retrospectively analyzed and they were collected during routine consultations. No patients were lost to follow-up.

### Clinical evaluation

Clinical data were collected during routine consultations in our department. In our practice, patients are clinically evaluated preoperatively and postoperatively at six and twelve months and, thereafter, annually. Our follow-up schedule consists of assessing pain and function using the American Orthopaedic Foot & Ankle Society (AOFAS) ankle and hindfoot scores, visual analogue scale (VAS) pain score, and the 12-Item Short Form Health Survey (SF-12) [9–13].

### Statistical analysis

The statistical analysis was performed by Matlab version 2008 (MathWorks, Natick, MA, USA). The statistical tests performed included ANOVA test and kappa test [14, 15]. For *k*-score, confidence intervals were defined at 95%. All statistical tests were considered significant with *p*-value < 0.05.

### Surgical technique

The patients were placed in the supine position on a radiolucent table with a thick pad under the ipsilateral hip to internally rotate the leg so that the toes pointed toward the ceiling of the room and a rigid board under the leg to support the alignment stand. The alignment stand was assembled before the patient installation. The non-operative limb was flexed at the knee and abducted at the hip to facilitate the lateral view with fluoroscopy.

**Figure 1.**
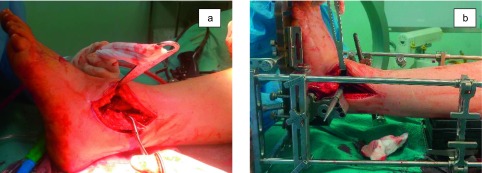
(a) The oblique fibular osteotomy and the reflection of the distal fibular fragment. (b) The correct internal rotation of the foot inside the alignment stand is checked. The lateral facet of the talus had to be parallel to the back end of the pointer.

We did not routinely use the tourniquet in order to avoid the postoperative pain related to the tourniquet. According to the original surgical technique, the incision should be straight starting just below the distal tip of the fibula and extending 15 cm proximally. Our surgical approach consisted of a 15-cm longitudinal incision along the lateral malleolus that curved under its tip to reach the sinus tarsi. A subperiosteal dissection of the fibula and of the anterior side of the tibia was made to have a full view of the joint line and of the anterior osteophytes. A release of the posterior capsule and ligaments was performed along the tibia and the fibula with a periosteal elevator. Then the anterior talofibular ligament was sectioned. At this point the fibular osteotomy was performed. The manufacturer’s surgical technique suggests to make an oblique osteotomy from superolateral to inferomedial starting 2.5–3 cm proximally to the joint line and ending 1.0–1.5 cm proximally to it. We performed a long oblique fibular osteotomy from superoposterior to inferoanterior starting 6–7 cm proximal to the joint line on the postero-lateral side of the fibula and ending 2 cm proximal to the joint line on the antero-lateral side of the malleolus (Figure 1a). This long osteotomy facilitates lengthening or shortening of the lateral malleolus to manage coronal deformities. The distal fibular segment was reflected distally to visualize the tibio-talar joint and fixed to the calcaneus with a 1.6 mm K-wire.

At this point a medial gutter release could be performed through a 3–4 cm incision medial to the anterior tibial tendon as suggested by the original surgical technique. We rarely performed the medial gutter release through this approach because it can be addressed later during the tibial preparation.

The anterior osteophytes were removed to allow for ankle ROM and to easily place the ankle in a neutral position. If the neutral position was not reached because of a plantar-flexion contracture we performed a percutaneous tendo-Achilles lengthening at this moment.

An oscillating saw was used on the lateral side of the distal tibia to create a flat lateral border between lateral tibia and talar surface: this is not described in the manufacturer’s surgical technique and it was helpful to smoothly position tibial and talar rails-drill guides later on. The medial/lateral talar dome was assessed with the sizer choosing the largest talar size possible while avoiding overhang.

Subsequently, the leg was placed in the alignment stand. The foot was internally rotated to obtain a mortise view. The correct internal rotation was checked, pushing the flat end of the pointer through the “Position” or “Talus” hole of the cutting guide: the lateral facet of the talus had to be parallel to the back end of the pointer (Figure 1b). Once the desired alignment was obtained, the foot was fixed to the footplate with a 4.0 mm transcalcaneal pin placed in a lateral to medial direction. The talus was then fixed to the footplate by a 4.0 mm pin placed in the neck as distal as possible under fluoroscopy control: this allowed for further correction of talar articular surface.

**Figure 2.**
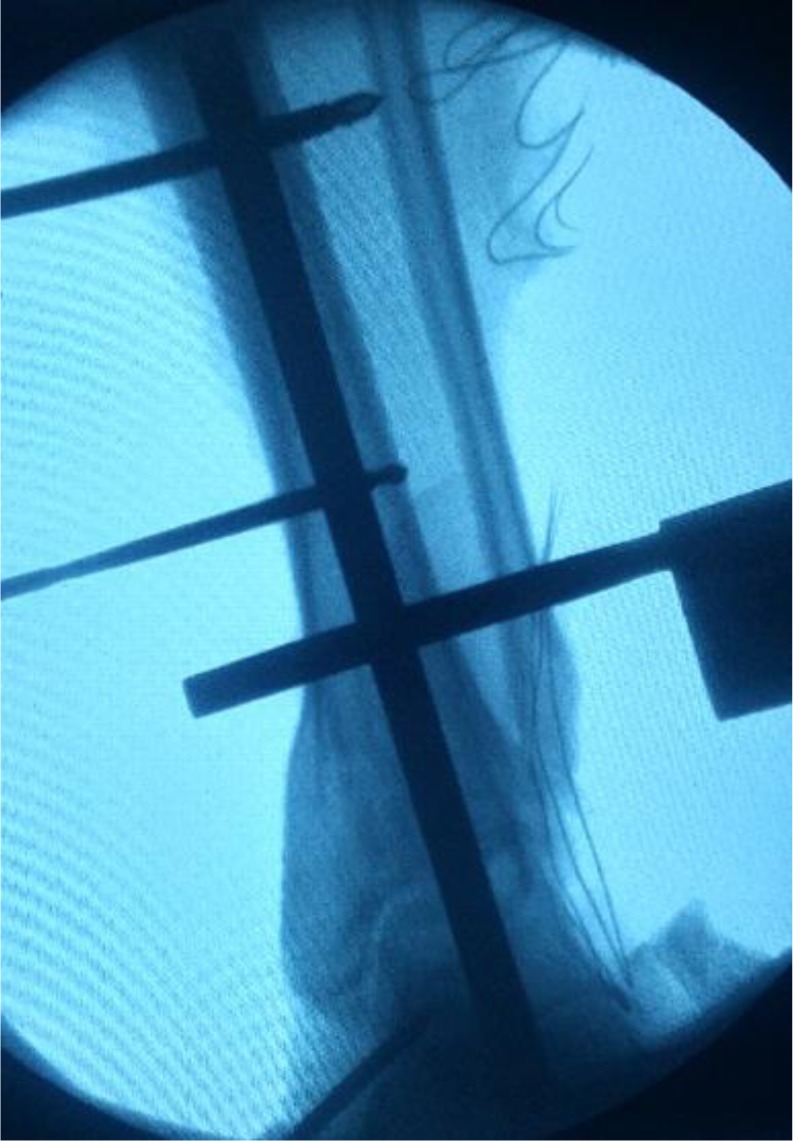
Alignment check. The pointer placed through the cutting guide must be parallel to the talar articular surface. The tibial alignment rod should be parallel to the mechanical axis of the tibia.

**Figure 3.**
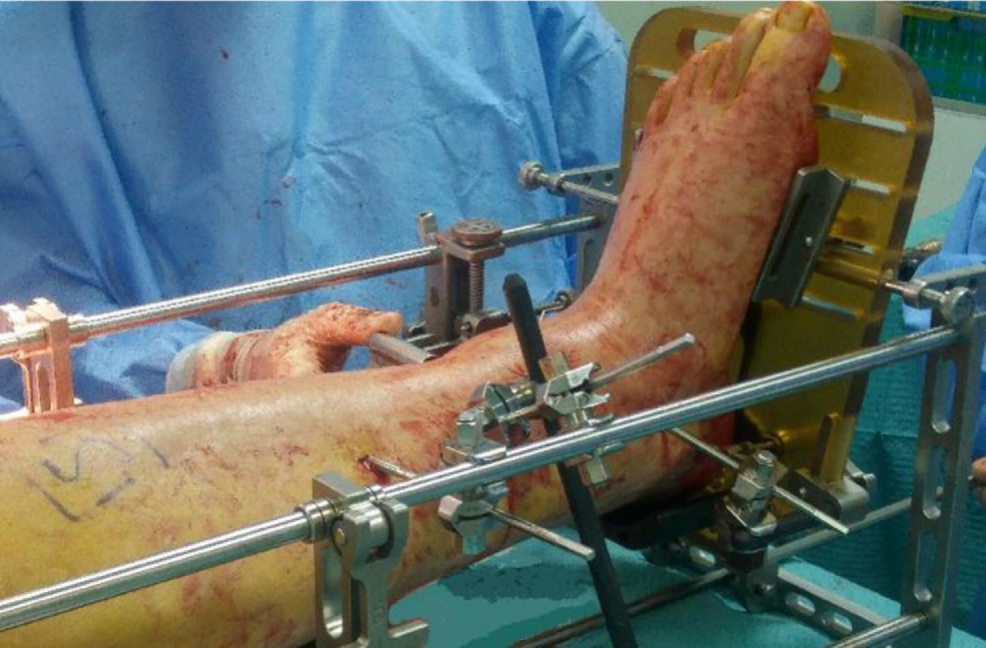
Tibial and talar pins. Standard positioning.

Fluoroscopic imaging was used to verify the tibial alignment: the tibial alignment rod should be parallel to the mechanical axis of the tibia. The pointer was placed through the “Position” hole of the cutting guide on the anterior surface of the ankle: it must be parallel to the talar articular surface (Figure 2). When the satisfactory position was achieved, the 5.0 mm tibial pins were placed on the medial side of the tibia: the assistant held the tibia anteriorly if an anterior sagittal talar malalignment was present. To enhance the rigidity of the fixation an adjunctive carbon fiber rod could be placed between the distal tibial pin and the lower medial rod of the stand (Figure 3).

Once the proper alignment was verified with the fluoroscopy, the pointer placed in the “Position” hole of the cutting guide of the selected size was used to check the level of the desired joint line in antero-posterior and proximal-distal sense. Then the amount of bone resection was assessed with the “Talus” and “Tibia #1” hole.

Bone removal was performed through the pre-cutting guide and, after the medial depth of the resection was established putting the contralateral talar provisional implant between the cutting guide and the drill bit, the definitive bone preparation was made through the “Talus” hole and the “Tibia #1” hole. “Tibia #2” hole was used to complete the remaining tibial resection, in particular on the medial side, and to remove osteophytes of the medial gutter.

**Figure 4.**
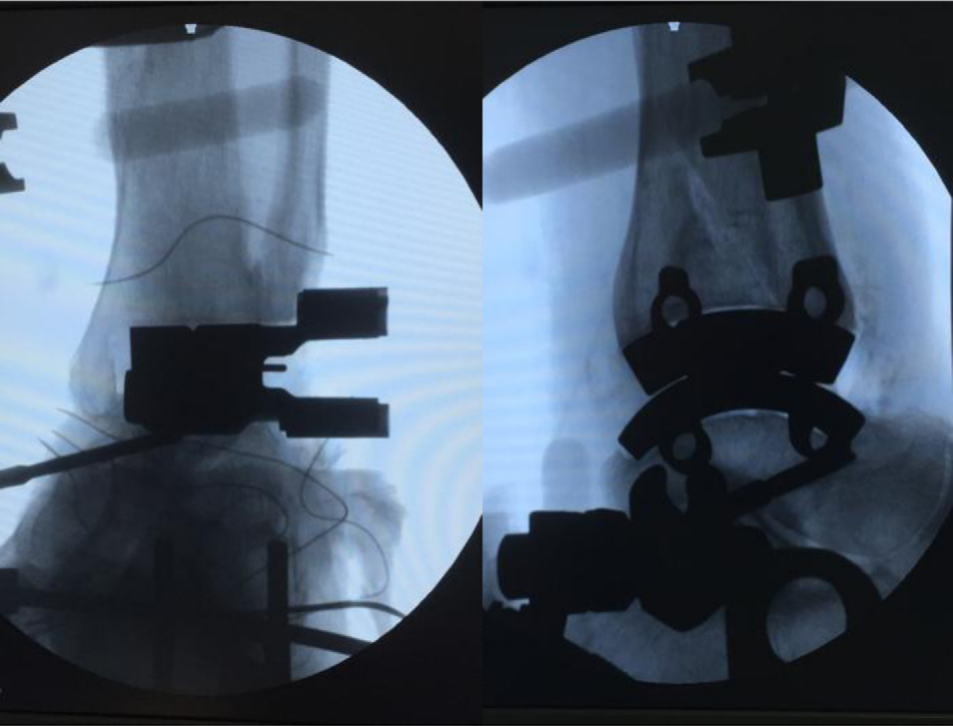
The rail hole drill guides are placed. In the antero-posterior view we confirm that they are medial enough to avoid lateral overhang of the implants. In the lateral view we confirm that they are flush along the talar and tibial cuts. The spreader pin is placed between the guides.

Then the rail hole drill guides were placed under antero-posterior fluoroscopy visualization to control that they were medial enough to avoid lateral overhang of the implants and in the lateral view to control that they were flush along the talar and tibial cuts after the tightest spreader pin was placed (Figure 4). After this, the rails were drilled through the rail holes and the trial placed to choose the correct liner and ensure correct position of the component in latero-medial and antero-posterior view. The final components were then inserted without cement fixation even if in the original surgical technique, the possibility to cement the implant was described.

In the manufacturer’s surgical technique, as the last step, the fibula is repaired with a plate. In our series, after fluoroscopic confirmation of proper alignment and ROM, the fibula was repaired with two or three 3.5 mm lag screws alone or, alternatively, with a plate if the amount of the fibular lengthening or shortening needed to correct deformation did not leave enough interfragmentary contact for screw fixation. The stability of the syndesmosis was tested with a hook and an additional syndesmosis screw fixation was done in the presence of a syndesmosis instability. A Broström repair [16] was performed for the anterior talofibular ligament before closing the wound.

### Postoperative care

**Figure 5.**
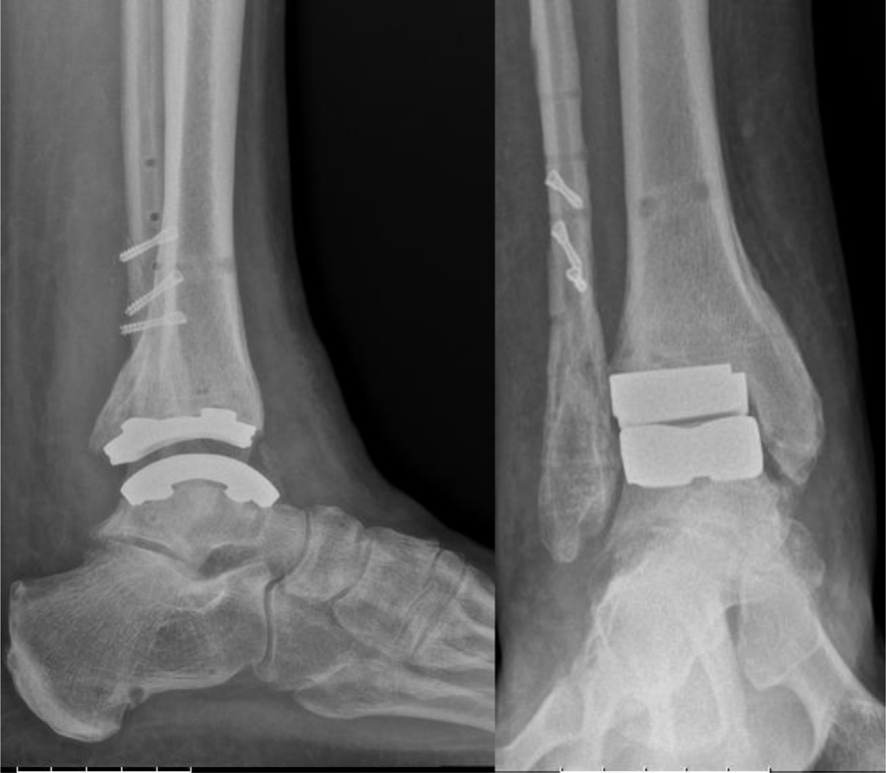
Lateral and antero-posterior view of the weight-bearing control radiographs.

Patients were placed into a short-leg cast and made non-weight-bearing for four weeks, then partial weight-bearing was allowed with a walker-boot for two weeks. Full weight-bearing and a rehabilitation program, which included stretching of the triceps surae, calf strengthening, and proprioceptive training, was started six weeks postoperatively (Figure 5).

## Results

There was a statistically significant increase in mean AOFAS hindfoot score from 32.8 preoperatively (*SD* 13.4, range 7–67) to 85.0 at the latest follow-up (*SD* 10.8, range 59–100, *p*-value < 0.001). Moreover, there was a statistically significant decrease in mean VAS pain score from 8.0 (*SD* 1.7, range 4–10) to 2.0 at the latest follow-up (*SD* 1.8, range 0–6, *p*-value < 0.001). In addition, there was a statistically significant increase in Physical and Mental Health Composite Scale scores (PCS and MCS) of the SF-12: PCS passed from a mean value of 30.2 preoperatively (*SD* 7.1, range 19.4–47.5) to 43.1 at the latest follow-up (*SD* 8.6, range 21.9–56.6, *p*-value < 0.001); MCS passed from a mean value of 44.6 (*SD* 7.9, range 23.5–67.8) to 53.5 at the latest follow-up (*SD* 7.2, range 35.0–65.2, *p*-value < 0.001).

Accessory concomitant procedures included: hardware removal (11), subtalar fusion (11), Achilles lengthening (8), double arthrodesis of talonavicular and subtalar joints (3), lateral calcaneal lengthening osteotomy (2), medial gutter debridement (2), peroneus longus to peroneus brevis transfer (1), distal tibial osteotomy (1), and triple arthrodesis (1). We considered the fibular osteotomy as part of the surgical technique and we did not include it among the accessory procedures even when a shortening or a lengthening of the lateral malleolus was needed to correct deformity.

We did not register loosening or malpositioning. There were four fibular plate removal and one syndesmotic fixation removal: three of them had delayed wound healing, one plate was removed because of patient discomfort, the syndesmotic screw was removed in consequence of mobilization.

## Discussion

We report the surgical technique we used performing 67 TARs with Zimmer TM TAR. We referred to the manufacturer’s surgical technique which we modified with some technical tips.

Firstly, the incision was curved distally toward the sinus tarsi. This provided a better view of the anterior joint line compared to the straight lateral incision, thus facilitating the removal of all the osteophytes that could prevent the achievement of the neutral position of the ankle. We believe that putting the ankle in neutral position before the bone resection is a fundamental step in order to reach a satisfactory ROM postoperatively.

We suggest a longer lateral fibular osteotomy in order to address any fibula-length issues and so a great number of joint coronal deformities. Furthermore, this long osteotomy allows a stable fixation with two or three lag screws placed from anterior to posterior and perpendicular to the osteotomy line. This can reduce the need for a plate removal consequent to a delayed wound healing or patient discomfort (even though this was the most frequent surgical procedure performed during follow-up). On the other hand, a longer fibular osteotomy makes the reflection of the malleolus more difficult because of strong adherences with the surrounding soft tissues.

We rarely performed the anteromedial incision for the medial gutter release. We noted that the bone preparation with the rotational burr in “Tibia #2” hole of the cutting guide most of the times provided an adequate cleaning of the medial gutter. We reserved the anteromedial incisions for cases in which osteophytes between the medial facet of talus and the lateral facet of medial malleolus were still present after “Tibia #2” cut and could cause medial impingement.

In our experience, the lateral border of the tibial plafond can interfere with the positioning of the rail-holes drill guide, even after an accurate bone preparation. To avoid this obstacle, we routinely smooth the lateral border of the tibia with an oscillating saw to create a flat surface between lateral tibia and talar surface: this is extremely helpful to smoothly position tibial and talar rails-drill guides.

In our series we did not cement the implants because a sufficient stability was reached at the end of the procedure. Moreover, the osseointegration characteristics of the trabecular metal assure a stable fixation over the time.

Finally, the functional and pain scores at a short-term follow-up are encouraging and are in line with the results of a previous paper describing early results of this prosthesis [7].

## Conclusions

Zimmer TM TAR is a relatively new implant designed that uses the lateral transfibular approach with the aim to reach a high degree of accuracy and reducing the variability of the positioning. We presented our surgical tips and the early results of this prosthetic design that are encouraging. We believe that they could be useful as an adjunct of the manufacturer’s surgical technique for surgeons that utilize these implants.

## Conflict of interests

The author(s) declare no potential conflicts of interest with respect to the research, authorship, and/or publication of this article.
